# Human cancer cells respond to cytosolic nucleic acids via enhanced production of interferon-β and apoptosis

**DOI:** 10.1186/2051-1426-3-S2-P250

**Published:** 2015-11-04

**Authors:** Shan Zhu, Yuan Qiao, Jing Wu, Guoxia Zang, Yong-Jun Liu, Jingtao Chen

**Affiliations:** 1 Institute of Translational Medicine, the First Hospital, Jilin University, Changchun, China; 2 MedImmune, Gaithersburg, Changchun, China; 3 Institute of Translational Medicine, the First Hospital, Jilin University, Changchun, Jilin, China

## 

The innate immune system utilizes pattern-recognition receptors (PRRs) to detect the invasion of pathogens and initiate host antimicrobial responses. Recently, it was reported that human cancer cells also expressed PRRs, and responded to cytosolic nucleic acids via the production of type I interferon and apoptosis in susceptible cells. However, the definite nucleic acids senses in different cancer cells remains unclear. In this study, we investigated the effects of nucleic acids in eight types of cancer cells, including pancreatic cancer, glioma, breast cancer, lung cancer, hepatoma, gastric cancer, colorectal cancer, and cervical cancer. We found that IFN-β secretion was increased after poly(I:C) and poly(dA:dT) stimulation. In order to understand the mechanism, we established the role of TLR3 and RIG-I/MDA-5 signaling in pancreatic cancer cell line PANC-1. poly(I:C) and poly(dA:dT) upregulated the expression of PKR, TLR3, RIG-I, MDA5 and LGP2. Knockdown experiments confirmed PKR, TLR3, TRIF, RIG-I, LGP2, and IPS-1 involved in response to cytosolic poly(I:C) and poly(dA:dT). In addition, poly(I:C) and poly(dA:dT) induced apoptosis via caspase-8 and caspase-9 in pancreatic cancer cells. In summary, cytosolic poly(I:C) and poly(dA:dT) induce IFN-β secretion via TLR3-TRIF and RIG-I/LGP2-IPS-1 signaling pathway and apoptosis in a caspase-dependent manner at cancer cells. This study may provide a novel way for drug and vaccine design on cancer immunotherapy.

